# Psychological factors associated with medication non-adherence after kidney transplant: a systematic review

**DOI:** 10.1186/s40359-026-05233-7

**Published:** 2026-07-27

**Authors:** Hui Ling Michelle Neo, Kuhanesan N. C. Naidu, Isabella Jia Hui Tang, Ho Yee Tiong, Shawn Yong Shian Goh, Cyrus Su Hui Ho

**Affiliations:** 1 Yong Loo Lin School of Medicine, National University of Singapore, Singapore, Singapore; 2 Department of Psychological Medicine, National University Hospital, Singapore, Singapore; 3 Saw Swee Hock School of Public Health, National University of Singapore, Singapore, Singapore; 4 Institute of Health and Wellbeing, Federation University Australia, Victoria, Australia; 5 National University Centre for Organ Transplantation, National University Hospital, Singapore, Singapore; 6 Health and Social Sciences Cluster, Singapore Institute of Technology, Singapore, Singapore

**Keywords:** Medications, Non-adherence, Kidney transplant, Self-reported, Psychological factors

## Abstract

Medication non-adherence remains a persistent and critical challenge among kidney transplant recipients, who demonstrate higher rates of non-adherence than other solid organ transplant populations. Given the associated risks of graft rejection, graft loss, and mortality, a clearer synthesis of underlying psychological factors is needed. This systematic review synthesized evidence on psychological factors associated with self-reported medication non-adherence among adult kidney transplant recipients, integrating quantitative and qualitative research to capture patients’ perspectives.

Following Joanna Briggs Institute guidelines, five electronic databases (Embase, PubMed, PsycINFO, Scopus, and Web of Science) were searched from inception to 5 May 2026. Eligible studies included quantitative and qualitative research involving adult recipients. Methodological quality was appraised using the Mixed Methods Appraisal Tool, and findings were integrated using a convergent integrated approach.

Forty-four studies comprising 11,180 participants were included. Non-adherence prevalence ranged from 12.0% to 89.2%. Four overarching themes were identified: psychological distress, perceptions and beliefs, coping strategies, and social-contextual factors. Depressive symptoms and low social support were the most consistent correlates. Qualitative findings highlighted emotional burden, stigma, and regimen complexity as additional barriers.

Medication non-adherence is multifactorial and context dependent. Integrating psychosocial screening and patient-reported outcomes into routine transplant care may improve adherence and long-term graft outcomes.

**Supplementary Information:**

The online version contains supplementary material available at https://doi.org/10.1186/s40359-026-05233-7.

## Introduction

Chronic Kidney Disease (CKD) is a life-threatening condition marked by irreversible loss of kidney function, leading to the accumulation of toxic metabolic waste and excess fluids in the bloodstream [1]. This progressive process results in widespread cellular, tissue, and organ damage, with common symptoms including fatigue, oedema, breathlessness, nausea, and poor appetite [1]. Kidney replacement therapies (KRT), namely dialysis and kidney transplantation, are required to maintain physiological stability in advanced disease [1, 2]. Most patients initiate dialysis while awaiting transplantation, although some undergo pre-emptive transplantation following close monitoring [2]. Kidney transplantation is widely recognised as the preferred treatment for CKD Stage 5, offering superior survival and quality-of-life outcomes compared with long-term dialysis [1, 2].

Following transplantation, all solid organ transplant recipients require lifelong immunosuppressive therapy to prevent acute and chronic rejection and prolong graft survival [2]. However, medication non-adherence, defined as any deviation in the initiation, implementation, or discontinuation of prescribed regimens [3], remains a significant challenge, particularly among kidney transplant recipients [4]. Evidence indicates that non-adherence is more prevalent in kidney transplantation than in other solid organ transplants [4]. A large meta-analysis of 147 studies involving over 29,000 recipients reported non-adherence rates of 35.6% in kidney recipients, compared with 14.5% in heart and 6.7% in liver transplant recipients [4]. Importantly, non-adherent kidney transplant recipients face substantially poorer outcomes, including a 2.66-fold higher risk of mortality, a 6.44-fold increased risk of graft loss, and a 2.28-fold higher risk of graft rejection [5]. These findings underscore the need to address medication adherence promptly globally.

Early kidney graft dysfunction is often asymptomatic, unlike liver or heart graft dysfunction, which may reduce kidney transplant recipients’ perceived need for strict medication adherence [6]. Additionally, graft loss in kidney transplantation is viewed as less immediately life-threatening due to the availability of dialysis as an alternative therapy [4]. In contrast, liver and heart transplant recipients have far fewer life-sustaining options [4]. Together, these factors highlight the organ-specific nature of medication adherence challenges in transplant care.

Research has extensively examined factors influencing medication non-adherence among adult kidney transplant recipients [7, 8]. A recent systematic review identified higher non-adherence risks among younger, male, unemployed patients, those with lower educational levels, living-donor transplant recipients, and individuals further from transplantation [9]. Psychosocial factors, including low social support, negative beliefs and behaviours, depression, and anxiety, were also strongly associated with non-adherence. Importantly, the authors recommended greater use of patient self-reports in future adherence research, emphasising the value of engaging patients as experts in understanding their own medication-taking behaviours.

Emerging research on heart [10, 11] and liver [12, 13] transplantation similarly highlights the growing importance of patient-reported outcomes (PROs). PROs capture patients’ own accounts of how they feel and function in relation to their condition and treatment, offering clinicians direct insight into patient perspectives [14]. Incorporating PROs can enhance patient-centred care and support more personalised treatment planning [14]. This trend supports a shift away from traditional adherence measures, such as serum drug levels, toward approaches that engage patients as authoritative sources of information about their medication adherence behaviours, including self-report tools and qualitative interviews.

Although the latest scoping review on medication non-adherence in kidney transplant recipients was conducted by Corr and colleagues in 2025 [15], its broad scope and the lack of critical appraisal of study quality limited the ability to draw definitive conclusions and to achieve a deeper understanding of patients’ perspectives in this field.

### Aim

Hence, this systematic review endeavours to address the identified gap in the literature by offering a timely and comprehensive overview of the psychological factors associated with self-reported medication nonadherence among adult kidney transplant recipients, providing updated insights into patients’ perspectives and experiences.

## Method

### Review design

This review followed the Joanna Briggs Institute (JBI) methodology for mixed methods systematic reviews, using a convergent integrated approach to synthesise qualitative and quantitative evidence within a single framework [16]. This approach was chosen because the review focused on psychological factors underlying self-reported medication non-adherence, which are experiential constructs measured quantitatively yet grounded in subjective experiences. Although most included studies were quantitative, they relied primarily on self-report psychometric instruments to assess psychological states rather than on objective clinical measures. These findings were therefore suitable for transformation into conceptual meaning units aligned with qualitative constructs. A convergent, integrated approach enabled the synthesis of statistical associations and experiential insights into a coherent whole, offering greater interpretive depth than parallel analyses. The review protocol was registered with PROSPERO (CRD42024575353).

### Search protocol, information sources, search strategy

Relevant studies were identified through a systematic search across five electronic databases: Embase (Elsevier), PubMed (NLM), PsycINFO (OvidSP), Scopus (Elsevier), and Web of Science (Thomson Reuters). The search covered publications from each database’s inception to 31 July 2025. To ensure the review remained current before publication, the search was updated to include studies published up to 5 May 2026. The search strategy was developed based on previous systematic reviews, input from the expert panel, and consultation with a research librarian. A broad keyword search was used to maximize sensitivity and maintain consistency across databases with different indexing systems (e.g., MeSH and Emtree). The final search combined terms related to kidney transplantation, psychological factors, and medication adherence, using the following structure: (renal transplant OR kidney transplant*) AND (depression OR anxiety OR transplant effects OR regret OR personality OR cognitive deficits OR cognitive impairment OR coping OR social support) AND (adherence OR compliance)*. Complete, database-specific search strategies are available in Supplementary File 1 (Table S1: Keyword Combination and Search Hits).

### Selection criteria

Eligible studies included quantitative research using standardised self-report questionnaires and qualitative interview-based studies involving adult kidney transplant recipients (≥ 18 years) who had undergone living- or deceased-donor transplantation. Excluded studies were reviews, meta-analyses, case studies, editorials, interventions, non-English publications, duplicate datasets, unavailable full texts, and studies with incomplete or non-combinable data. Independent reviewers conducted study selection according to predefined eligibility criteria, and the PRISMA 2020 guidelines (Supplementary File 2) were used to guide transparent reporting of the review process [17]. Screening was conducted independently by two reviewers (KN and IT), with disagreements resolved by a third reviewer (MN).

### Quality assessment of included studies

All included studies were appraised using the Mixed Methods Appraisal Tool (MMAT) [18, 19], which evaluates qualitative, quantitative, and mixed methods studies within a single framework. Consistent with MMAT guidance, no overall quality scores were assigned. Methodological quality was assessed across relevant domains, with independent appraisals by KN and IT, discrepancies resolved with MN, and results reported in Table 1.

**Table 1:** Quality assessment of included studies using MMAT

References	Screening		1. Qualitative					2. Quantitative RCT					3. Quantitative non-randomised					4. Quantitative descriptive					5. Mixed methods				
	S1	S2	1.1	1.2	1.3	1.4	1.5	2.1	2.2	2.3	2.4	2.5	3.1	3.2	3.3	3.4	3.5	4.1	4.2	4.3	4.4	4.5	5.1	5.2	5.3	5.4	5.5
Alkatheri et al. 2016 [20]	Y	Y											N	Y	Y	Y	Y										
Balakrishnan et al. 2022 [21]	Y	Y											N	Y	Y	Y	Y										
Bünemann et al. 2020 [22]	Y	Y											N	Y	Y	Y	Y										
Burkhalter et al. 2013 [23]	Y	Y											N	Y	N	Y	Y										
Cardol et al. 2023 [24]	Y	Y											N	Y	Y	N	Y										
Chisholm-Burns et al. 2010 [25]	Y	Y											N	Y	Y	N	Y										
Cukor et al. 2009 [26]	Y	Y											N	Y	Y	N	Y										
de Boer et al., 2020 [27]	Y	Y											N	Y	Y	N	Y										
De Brito et al. 2015 [28]	Y	Y											N	Y	Y	Y	Y										
Demian et al. 2016 [29]	Y	Y											N	Y	Y	N	Y										
Derejie et al. 2025 [30]	Y	Y											N	Y	Y	N	Y										
Gelb et al. 2010 [31]	Y	Y											N	Y	Y	N	Y										
Griva et al. 2012 [32]	Y	Y											N	Y	Y	Y	Y										
Griva et al. 2018 [33]	Y	Y											N	Y	Y	Y	Y										
Jana et al. 2014 [34]	Y	Y											N	Y	Y	Y	Y										
Karazeybek 2025 [35]	Y	Y											N	Y	Y	N	Y										
Kim et al. 2016 [36]	Y	Y											N	Y	Y	Y	Y										
Klewitz et al. 2019 [37]	Y	Y											N	Y	Y	Y	Y										
Kostalova et al. 2021 [38]	Y	Y											N	Y	Y	N	Y										
Lalic et al. 2014 [39]	Y	Y											N	Y	Y	Y	Y										
Liu et al. 2021 [40]	Y	Y											N	Y	Y	Y	Y										
Low et al. 2016 [41]	Y	Y	Y	Y	Y	Y	Y																				
Massey et al. 2015 [42]	Y	Y											N	Y	Y	N	Y										
Memory et al. 2022 [43]	Y	Y	Y	Y	Y	Y	Y																				
Muduma et al. 2016 [44]	Y	Y	Y	Y	Y	Y	Y																				
Neubert, et al. 2021 [45]	Y	Y											N	Y	Y	Y	Y										
Nöhre et al. 2026 [46]	Y	Y											N	Y	Y	Y	Y										
Paterson et al. 2018 [47]	Y	Y											N	Y	Y	Y	Y										
Patzer et al. 2016 [48]	Y	Y											N	Y	Y	N	Y										
Pisanti et al. 2016 [49]	Y	Y											N	Y	Y	Y	Y										
Reber et al. 2016 [50]	Y	Y											N														
Saravanakumar et al. 2019 [51]	Y	Y											N	Y	Y	Y	Y										
Scheel et al. 2018 [52]	Y	Y											N	Y	Y	Y	Y										
Scheel et al. 2019 [53]	Y	Y											N	Y	Y	N	Y										
Teng et al. 2015 [54]	Y	Y											N	Y	Y	Y	Y										
Uyar et al. 2022 [55]	Y	Y											N	Y	Y	N	Y										
Vankova et al. 2018 [56]	Y	Y																					Y	Y	Y	Y	Y
Villeneuve et al. 2020 [57]	Y	Y											N	Y	Y	Y	Y										
Weng et al. 2013 [58]	Y	Y											N	Y	Y	N	Y										
Yang et al. 2022 [59]	Y	Y											N	Y	Y	N	Y										
Zachciał et al. 2022 [60]	Y	Y											N	Y	Y	N	Y										
Zachciał et al. 2022 [61]	Y	Y											N	Y	Y	N	Y										
Zhang et al. 2021 [62]	Y	Y											N	Y	Y	Y	Y										
Zhou et al. 2025 [63]	Y	Y											N	Y	Y	Y	Y										

*Abbreviation: CT* Can’t tell, *N* No, *RCT* Randomised control trial, *Y* Yes

S1. Are there clear research questions?

S2. Do the collected data allow for addressing the research questions?

1.1. Is the qualitative approach appropriate to answer the research question?

1.2. Are the qualitative data collection methods adequate to address the research question?

1.3. Are the findings adequately derived from the data?

1.4. Is the interpretation of results sufficiently substantiated by data?

1.5. Is there coherence between qualitative data sources, collection, analysis and interpretation?

2.1. Is randomization appropriately performed?

2.2. Are the groups comparable at baseline?

2.3. Are there complete outcome data?

2.4. Are outcome assessors blinded to the intervention provided?

2.5 Did the participants adhere to the assigned intervention?

3.1. Are the participants representative of the target population?

3.2. Are measurements appropriate regarding both the outcome and intervention (or exposure)?

3.3. Are there complete outcome data?

3.4. Are the confounders accounted for in the design and analysis?

3.5. During the study period, is the intervention administered (or exposure occurred) as intended?

4.1. Is the sampling strategy relevant to address the research question?

4.2. Is the sample representative of the target population?

4.3. Are the measurements appropriate?

4.4. Is the risk of nonresponse bias low?

4.5. Is the statistical analysis appropriate to answer the research question?

5.1. Is there an adequate rationale for using a mixed methods design to address the research question?

5.2. Are the different components of the study effectively integrated to answer the research question?

5.3. Are the outputs of the integration of qualitative and quantitative components adequately interpreted?

5.4. Are divergences and inconsistencies between quantitative and qualitative results adequately addressed?

5.5. Do the different components of the study adhere to the quality criteria of each tradition of the methods involved?

### Data extraction and synthesis

Data extraction was conducted independently by two reviewers (KN and MN) using a JBI-standardised form adapted for mixed methods reviews. Extracted data included publication details, study characteristics, sample demographics, measurement instruments, and key findings related to medication non-adherence (Table 2). Owing to substantial heterogeneity in psychological measures and outcome definitions, a meta-analysis was not undertaken [17–19]. Qualitative data were extracted as thematic statements or participant quotations, accompanied by interpretations. Quantitative findings that were both significant and non-significant were extracted, considered, and qualitised by transforming into thematic meaning units reflecting underlying psychological constructs (e.g., emotional distress undermining treatment engagement). Integration prioritised effect direction, consistency, and conceptual relevance over statistical magnitude, with quantitative findings coded inductively alongside qualitative data using a shared analytic framework.

**Table 2:** Detailed characteristics of all the included studies

Study Reference	Country	Study Design	Sample Size	Age (M ± SD; Median (Q25-Q75))	Psychological Measures	Adherence Measures	Non-adherence Rates (defined as “less than perfect” adherence)	Main Findings (related to medication non-adherence)
Alkatheri et al. 2016 [20]	Saudi Arabia	Cross-sectional	151	46.7 ± 16.5	TSQM 1.4	MMAS-8	66.0%	Lower treatment satisfaction (in the convenience and side effects domains).
Balakrishnan et al. 2022 [21]	United States of America	Cross-sectional	234	51.0 (21.0–74.0)	Health Literacy 3-item screener, PROMIS depression short form	ASK-12, drug levels	28.0%	Lower health literacy, higher depressive symptoms.
Bünemann et al. 2020 [22]	Germany	Cross-sectional	570	51.9 ± 14.17	BMQ	MARS-D	40.2%	Lower perceived benefits, greater medication concerns.
Burkhalter et al. 2013 [23]	Switzerland	Cross-sectional	926	59.7 (50.26–67.77)	ESS, STCS-DS, DASS-21	BAASIS	35.0%	Higher daytime sleepiness score, higher depressive symptoms.
Cardol et al. 2023 [24]	Netherlands	Cross-sectional	460	58.5 ± 12.5	SF-36, PHQ-ADS	SMAQ	69.8%	Higher depressive symptoms, higher anxiety symptoms.
Chisholm-Burns et al. 2010 [25]	United States of America	Cross-sectional	61	48.9 ± 11.44	MSSS-5	ITAS	Very low (mean score 11.1 ± 2.29/12.0)	Lower affectionate support, lower instrumental support, high forgetfulness.
Cukor et al. 2009 [26]	United States of America	Cross-sectional	94	44.1 ± 12.2	BDI, MHLC	ITAS	40%	Higher depressive symptoms.
de Boer et al., 2020 [27]	Germany	Cross-sectional	72	52.4 ± 14.2	IKQ	MARS-D	Very low (mean score 24.4 ± 1.2/25.0)	No significant association with health literacy.
De Brito et al. 2015 [28]	Brazil	Cross-sectional, comparative	25	47.6 ± 13.6	LSSI, WCS	BAASIS	Not applicable	Higher symptoms of stress, higher palliative coping pattern (religion/fantasy thoughts and emotion-focused coping).
Demian et al. 2016 [29]	Canada	Cross-sectional	96	52.8 ± 12.5	HLQ	TxEQ-Adherence subscale	75.0%	Lower health literacy.
Derejie et al. 2025 [30]	Ethiopia	Cross-sectional	270	37.0 (28.0–45.0)	SMAQ	SMAQ, BAASIS	18.1%	Higher forgetfulness.
Gelb et al. 2010 [31]	Canada	Cross-sectional	103	50.1 ± 12.4	EPS Test, CES-D	TxEQ-Adherence subscale	Very low (mean score 20.8 ± 3.6/25.0)	Cognitive impairment, higher number of depressive symptoms.
Griva et al. 2012 [32]	United Kingdom	Cross-sectional	218	49.6 ± 12.3	BDI-II, TxEQ- Worry, Guilt, Perceived Responsibility subscales, BMQ, KDQoL-SF v1.3	MARS, drug levels	51.4%	Dialysis vintage, doubts about necessity, and lower worry about the viability of the graft explained 16.1–36.0% of self-reported non-adherence. Higher depressive symptoms were related only to intentional non-adherence.
Griva et al. 2018 [33]	Singapore	Cross-sectional	152	49.5 ± 11.4	TxEQ, BMQ, MSPSS, DASS-21	MARS	19.7%	Intentional non-adherence was predicted by higher medication concerns. Overall non-adherence was predicted by a deliberate decision-making process involving the weighing of the costs of using immunosuppressive drugs against their perceived benefits.
Jana et al. 2014 [34]	India	Cross-sectional, comparative	105	35.0 ± 9.8	HADS, WHOQOL-BREF	MMAS-8	Not reported	Neither depressive nor anxiety symptoms were associated with non-adherence.
Karazeybek 2025 [35]	Turkey	Cross-sectional	168	42.2 ± 12.5	MSPSS	IMUAS	Low (mean score 50.2 ± 3.7/55.0)	Lower total, family, and friend social support.
Kim et al. 2016 [36]	Korea	Cross-sectional	105	46.9 ± 11.8	SF-36	TxEQ-Adherence subscale	Very low (mean score 4.1 ± 0.9/5.0)	Lower mental health and well-being.
Klewitz et al. 2019 [37]	Germany	Cross-sectional	397	51.0 ± 15.0	HADS-D, F-SozU K7, MESI, SIMS-D	MARS-D	39.8%	Lower levels of satisfaction with immunosuppressant medication information.
Kostalova et al. 2021 [38]	Czech Republic	Cross sectional, follow-up	134	58.9 (47.6–67.9)	BMQ-CZ	MARS-CZ, BAASIS	12.0%	Higher medication concerns.
Lalic et al. 2014 [39]	Serbia	Cross-sectional, comparative	60	44.5 ± 11.4	SMAQ	SMAQ, drug levels	28.3%	Higher forgetfulness.
Liu et al. 2021 [40]	China	Cross-sectional	293	42.5 ± 10.0	RESE, BMQ	BAASIS	23.1%	Lower perceived self-efficacy in managing anger-irritation, despondency-distress and positive affect. Higher medication concerns, lower medication necessity.
Low et al. 2016 [41]	Australia	Cross-sectional, qualitative	25	57.0 ± 14.5	-	-	NR	Providing regular reminders about the lifestyle benefits of kidney transplantation, creating opportunities for patients to share their experiences, and promoting the use of a reminder alarm to take medications will reduce the stress of managing medication after a kidney transplant.
Massey et al. 2015 [42]	Netherlands	Cross-sectional, comparative	62	25.6 ± 2.9	BPNS, COPE, CLQ	PANAS, SWLS, BAASIS	65.0%	No significant difference between adherent and non-adherent patients on positive affect or satisfaction with life. There was a trend for patients classified as adherent to score higher on negative affect than those classified as nonadherent.
Memory et al. 2022 [43]	United Kingdom	Cross-sectional, qualitative	11	55.0 (33.0–77.0)	-	-	NR	Gathering health-related knowledge helps with medical management (which includes adherence to complex medication regimens). The combined burden of symptoms, medication side effects, underlying kidney condition, and comorbidities were considered to limit engagement with medical self-management.
Muduma et al. 2016 [44]	United Kingdom	Cross-sectional, qualitative	40	50.1 (29.0–68.0)	-	-	NR	Non-adherence was mainly unintentional, driven by forgetfulness, disruptions to routine, pill burden, and difficulties integrating medication schedules into daily life.
Neubert, et al. 2021 [45]	Germany	Cross-sectional	56	56.7 ± 11.5	QMI, SSQ, SF-8	MESI	Moderate (mean score 15.0 ± 6.15/33.0, with a cut-off being 15.0)	For male recipients, lower social support, poorer marital relationship quality, and lower mental and physical quality of life. For female recipients, there is a lower mental quality of life.
Nöhre et al. 2026 [46]	Germany	Cross-sectional	838	52.3 ± 13.5	BMQ, HADS, F-SozU K7, SF-12	BAASIS	22.1%	Lower mental quality of life, higher depressive symptoms, higher anxiety symptoms, lower perceived social support, and lower medication necessity.
Paterson et al. 2018 [47]	Canada	Cross-sectional	211	53.9 ± 11.9	CES-D, SES, GSE, NGSE, ASES, MASES-R, EPS	TxEQ-Adherence subscale, drug levels	Very low (mean score 21.3 ± 3.8/25.0)	Everyday problem solving and self-efficacy had direct positive associations with adherence. Depressive symptoms were negatively associated with self-efficacy, but not adherence.
Patzer et al. 2016 [48]	United States of America	Cross-sectional	99	52.5 ± 13.2	REALM, MMSE	PMAQ, drug levels	35.0%	Lower health literacy.
Pisanti et al. 2016 [49]	Italy	Cross-sectional	174	52.3 ± 13.1	TRS, CISS-SV, SCL	TxEQ-Adherence subscale	Very low (mean score 4.5 ± 0.7/5.0)	Higher avoidance-oriented coping.
Reber et al. 2016 [50]	Germany	Cross-sectional	74	54.1 ± 12.8	MESI, HADS	BAASIS	24.3%	Negative medication beliefs, higher depressive symptoms, and higher anxiety symptoms.
Saravanakumar et al. 2019 [51]	India	Cross-sectional	279	36.0 ± 9.0	ITBS	ITAS	26.5%	Higher perceived barriers.
Scheel et al. 2018 [52]	Germany	Cross-sectional	330	52.9 ± 13.8	HADS-D, F-SozU K7, SF-12, MESI	BAASIS	33.6%	Poorer social support, lower mental quality of life, and higher physical quality of life. There was no association with depression and anxiety.
Scheel et al. 2019 [53]	Germany	Cross-sectional	267	52.8 ± 13.7	TxEQ, SF-12	BAASIS, drug levels	33.3%	Higher worry about transplant.
Teng et al. 2015 [54]	China	Cross-sectional	231	44.9 ± 10.8	SEISES	AIMS	89.2%	Higher level of symptom distress.
Uyar et al. 2022 [55]	Turkey	Cross-sectional	260	45.4 (46.0–64.0)	DASS-21	ITAS	Moderate (mean score 7.0 ± 0.9/12.0)	Higher depressive, anxiety, and stress symptoms.
Vankova et al. 2018 [56]	Czech Republic	Cross-sectional, mixed methods	211	55.0 ± 12.4	BMQ-CZ	MARS-CZ, drug levels	18.0%	Higher medication concerns, lower medication necessity.
Villeneuve et al. 2020 [57]	France	Prospective cohort	712	51.1 ± 13.1 and 54.5 ± 13.6	SF-36	MMAS-4, drug levels	15.0%	More depression episodes, poorer mental quality of life.
Weng et al. 2013 [58]	United States of America	Cross-sectional	252	54.7 (44.6–62.9)	ITBS, HADS, ISEL-12, PSS-4, sTOFHLA	ITAS	40.9%	Higher perceived barriers.
Yang et al. 2022 [59]	China	Cross sectional, follow-up	246	43.5 ± 11.5	BMQ, MSPSS, TPB	BAASIS	39.4%	Lower intentions to take the medications.
Zachciał et al. 2022 [60]	Poland	Cross-sectional	190	61.7 ± 12.1	WHOQoL-BREF, MMSE, HADS, AIS, TFI	BAASIS, drug levels	19.0%	Higher anxiety symptoms, and poorer cognitive functions.
Zachciał et al. 2022 [61]	Poland	Cross-sectional	210	59.9 ± 13.2	WHOQoL-BREF, MMSE, HADS, AIS, TFI	ARMS, drug levels	25%	Lower acceptance of illness. Higher levels of frailty.
Zhang et al. 2021 [62]	China	Cross-sectional	1357	41.3	TPB, HBM	BAASIS	33.5%	Higher perceived barriers and higher perceived seriousness.
Zhou et al. 2025 [63]	China	Cross-sectional	351	47.4 ± 11.2	MLS-C, SSRS, BMQ, RESE-C	BAASIS	37.6%	Lower medication literacy, lower social support, negative medication beliefs, lower perceived regulatory emotional self-efficacy.

*AIMS* Adherence with Immunosuppressive Medication Scale, *AIS* Acceptance of Illness Scale, *ARMS* Adherence to Refills and Medications Scale, *ASES* Adherence Self-Efficacy Scale, *ASK-12* Adherence Starts with Knowledge 12, *BAASIS* Basel Assessment of Adherence Scale, *BDI(-II)* Beck Depression Inventory (II), *BMQ(-CZ)* Beliefs about Medicines Questionnaire (-Czech version), *BPNS* Basic Psychological Needs Scale, *CES-D* Center for Epidemiological Studies Depression Scale, *CISS-SV* Coping Inventory for Stressful Situations Shortened Version, *CLQ* Course of Life Questionnaire, *COPE* Coping Orientation to Problem Experienced, *DASS-21* Depression, Anxiety, and Stress Scale 21-item, *ESRD-SCL-TM* End-Stage Renal Disease Symptom Checklist- Transplant Module, *EPS* Everyday Problem Solving, *ESS* Epworth Sleepiness Scale, *F-SozU K7* German Perceived Social Support Questionnaire, *GSE* General Self-Efficacy Scale, *HADS(-D)* Hospital Anxiety and Depression Scale (- German Version), *HBM* Health Belief Model questionnaire, *HLQ* Health Literacy Questionnaire, *IIRS* Illness Intrusiveness Rating Scale, *IKQ* Immunosuppressant Knowledge Questionnaire, *IMUAS* Immunosuppressive Medication Use Adherence Scale, *ISEL-12* Interpersonal Support and Evaluation List-12, *ITAS* Immunosuppressant Therapy Adherence Scale, *ITBS* Immunosuppressant Therapy Barriers Scale, *KDQoL-SF v1.3* Kidney Disease Quality of Life Short Form version 1.3, *LSSI* Lipp Stress Symptoms Inventory, *MARS(-CZ; -D)* Medication Adherence Report Scale (- Czech version; -German version), *MASES-R* Medication Adherence Self-Efficacy Scale—Revised, *MESI* Medication Experiences Scale for Immunosuppressants, *MHLC* Multidimensional Health Locus of Control Scales, *MLS (-C)* Medical Literacy Scale (-Chinese version), *MMAS (−4, −8)* Morisky Medication Adherence Scale, *MMSE* Mini-Mental State Examination, *MSSS-5* Modified Social Support Survey, *MSPSS* Multidimensional Scale of Social Support, *NGSE* New General Self-Efficacy Scale, *PANAS* Positive And Negative Affect Schedule, *PHQ-ADS* Patient Health Questionnaire Anxiety and Depression Scale, *PMAQ* Patient Medication Adherence Questionnaire, *PSS-4* Perceived Stress Scale-4, *PROMIS Depression Short Form* Patient Reported Outcomes Measurement Information System Depression Short Form, *QMI* Quality of Marriage Index, *REALM* Rapid Assessment of Adult Literacy in Medicine, *RESE (-C)* Regulatory Emotional Self-Efficacy (-Chinese version), *SCL* Symptom Checklist, *SEISES* Symptom Experience of Immunosuppressive-related Side Effects Scale, *SES* Self-Efficacy Scale, *SF(−8, −12, −36)* Short-Form Health Survey (−8 items, − 12 items, − 36 items), *SIMS(-D)* Satisfaction with Information about Medicines Scale (-German version), *SMAQ* Simplified Medication Adherence Questionnaire, *SSRS* Social Support Rating Scale, *SSQ* Social Support Questionnaire, *STCS-DS* Swiss Transplant Cohort Study Daytime Sleepiness single-item scale, *sTOFHLA* Short Test of Functional Health Literacy in Adults, *SWLS* Satisfaction with Life Scale, *TFI* Tilburg Frailty Indicator, *TPB* Theory of Planned Behavoiur Questionnaire, *TRS* Transplant Related Stressors, *TSQM 1.4* Treatment Satisfaction Scale for Medication Version 1.4, *TxEQ* Transplant Effects Questionnaire, *WCS* Ways of Coping Scale, *WHOQOL-BREF* World Health Organization Quality of Life Scale

Methodological quality informed the integration by prioritising themes supported by higher-quality and consistent studies. Quantitative and qualitative findings were merged into a single dataset and thematically synthesised to address the review objective. The final synthesis presents a unified line of evidence on psychological factors associated with self-reported medication non-adherence among adult kidney transplant recipients.

## Results

### Search outcomes

**Fig. 1:**
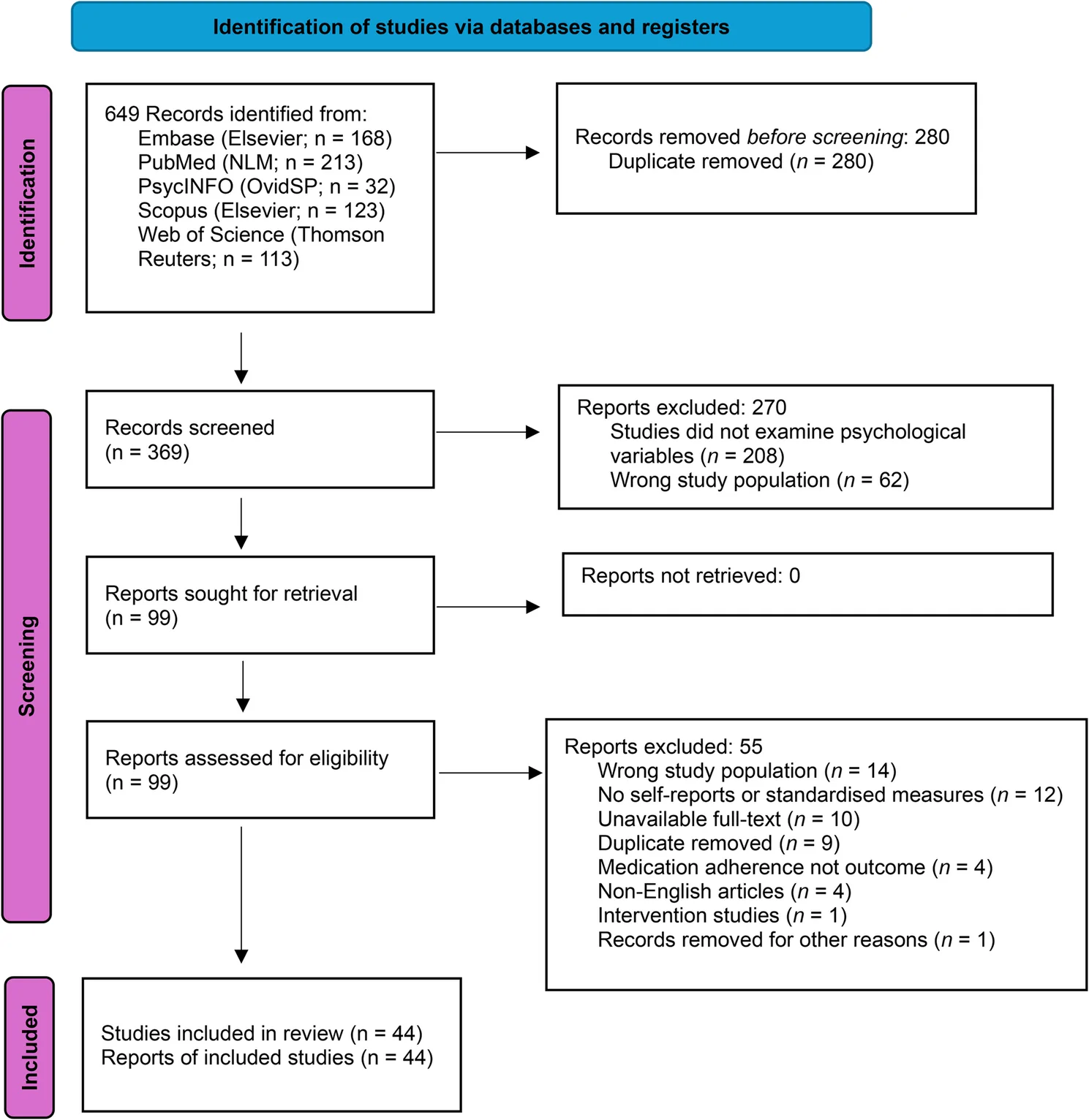
PRISMA diagram

The database search across five electronic databases yielded 649 records. After removing 280 duplicates, 369 records remained for screening. Of these, 270 were excluded based on titles and abstracts. A full-text review of 99 articles led to the exclusion of 55, leaving 44 studies included. The study selection process is detailed in the PRISMA diagram (Fig. 1).

### Quality appraisal

Critical appraisal using the MMAT [64, 65] showed most studies met screening and design-specific criteria, indicating a generally robust evidence base. Common limitations included inadequate control of confounders and limited representativeness, resulting in a moderate risk of bias. While findings are credible for synthesis, cautious interpretation is warranted, especially for studies examining anxiety and cognitive functioning.

### Study characteristics

In total, 44 articles [20–63] encompassed 11,180 participants, with individual sample sizes ranging from *N* = 25 to 1357, and mean participant ages ranging from 25.6 to 61.7 years. The studies were conducted across 21 countries and can be grouped by region as follows. Europe contributed the largest number of studies (*n* = 21), followed by Asia (*n* = 12), North America (*n* = 8). Oceania was represented by Australia (*n* = 1), while South America and Africa were each represented by one study from Brazil (*n* = 1) and Ethiopia (*n* = 1), respectively. The most frequently investigated factor was depressive symptoms, followed by beliefs about medications, anxiety symptoms, and health literacy. Detailed characteristics of the included studies and the review findings are presented in Table 2.

### Prevalence of medication-non-adherence

Medication non-adherence rates varied widely, ranging from 12.0% [39] to 89.2% [54]. Several studies reported non-adherence rates exceeding 50.0% [20, 24, 29, 42], underscoring the persistence of this issue.

### Integration of findings

**Fig. 2:**
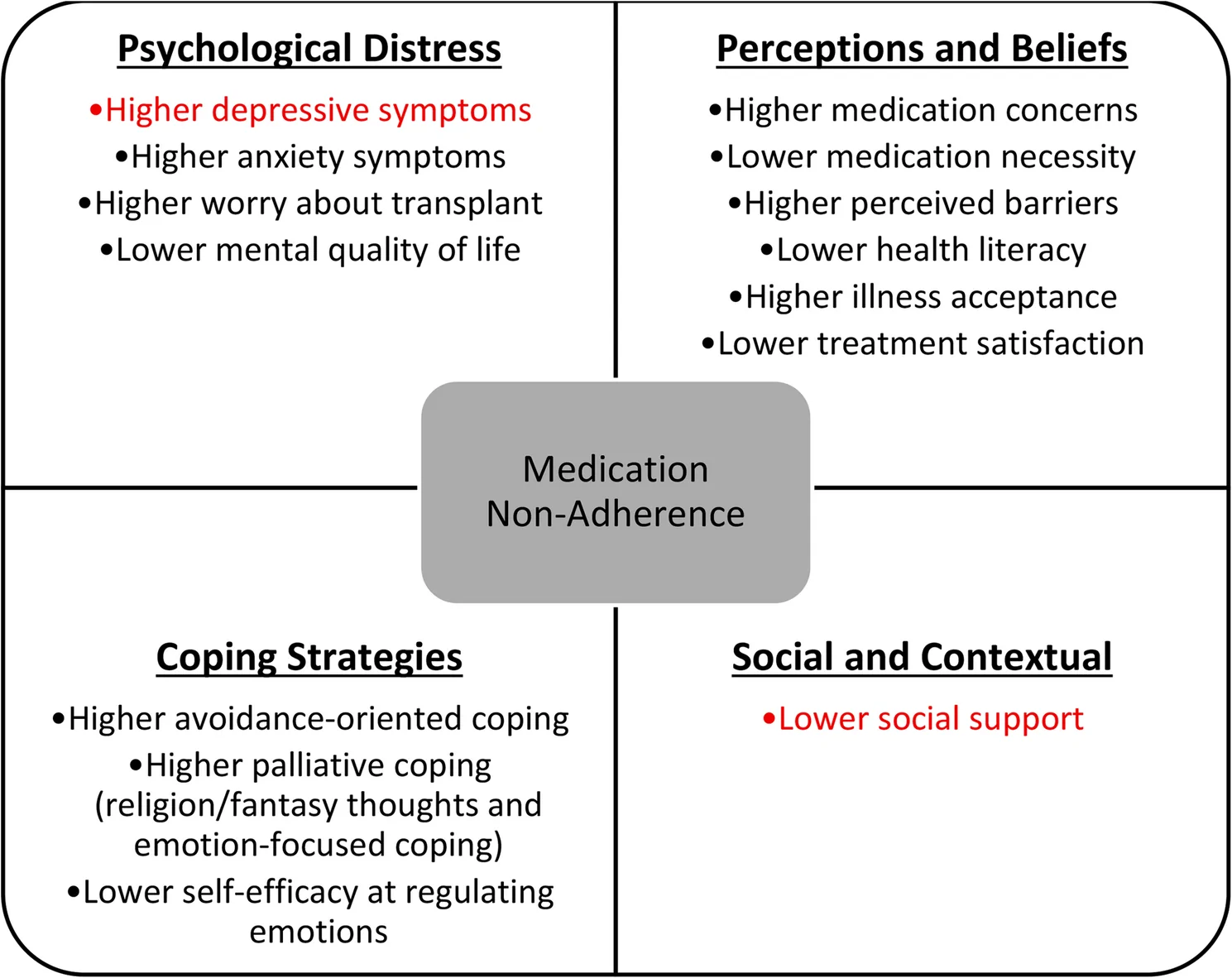
Psychological factors related to self-report medication non-adherence in adult kidney transplant recipients were classified into four main themes. Under theme (1) psychological distress, factors include higher depressive symptoms, higher anxiety symptoms, higher worry about transplant, and lower mental quality of life. Under theme (2) perceptions and beliefs, factors include higher medication concerns, lower medication necessity, higher perceived barriers, lower health literacy, higher illness acceptance, and lower treatment satisfaction. Under theme (3) coping strategies, factors include higher avoidance-oriented coping, higher palliative coping (religion/fantasy thoughts and emotion-focused coping), and lower self-efficacy at regulating emotions. Under theme (4) social and contextual, factors include lower social support. Factors supported by the strongest and most consistent evidence across included studies are highlighted in red. Note. Red indicates factors supported by the strongest and most consistent evidence across included studies

The integration of both quantitative and qualitative synthesis led to the identification of four main themes related to medication non-adherence in adult kidney transplant recipients: (1) psychological distress, (2) perceptions and beliefs, (3) coping strategies, and (4) social and contextual factors, as illustrated by Fig. 2.

#### Psychological distress

##### Depressive symptoms

Depressive symptoms emerged as a prominent psychological correlate of medication non-adherence across studies from multiple countries, including the United States, Europe, and Turkey [21, 23, 24, 26, 29, 55]. Higher levels of depression were consistently associated with greater non-adherence. Some evidence suggests that physiological features of depression, such as fatigue and sleep disturbances, may disrupt routine medication-taking behaviours [23]. Notably, depressive symptoms were linked more strongly to intentional non-adherence than unintentional non-adherence, indicating that mood-related cognitive and motivational factors may influence deliberate decisions to deviate from prescribed regimens [32].

##### Anxiety symptoms

Anxiety symptoms [24, 55] and higher worry about the transplanted kidney and health [53] were also positively correlated with higher medication non-adherence. In contrast, some studies [34, 52] did not find such associations with psychological distress, suggesting that contextual or methodological differences may be at play.

##### Cognitive functioning and mental quality of life

Several studies have identified cognitive functioning and mental health as key factors affecting non-adherence. Non-adherent patients showed poorer problem-solving ability [31, 47], greater depressive [31], anxiety symptoms and cognitive impairment [60], and lower mental quality of life, highlighting the influence of cognitive and psychological well-being on medication-taking behaviours. Meanwhile, studies using quality of life measures (e.g., SF-36, WHOQoL-BREF) frequently identified lower mental quality of life as a key barrier to adherence [36, 52, 57].

#### Perceptions and beliefs

##### Beliefs about medications

Beliefs about medications, including both necessity and concerns, were another recurring factor. Studies [22, 33, 38, 40, 56] using the Beliefs about Medicines Questionnaire (BMQ) have consistently shown that greater medication concerns and lower perceived necessity consistently affect non-adherence.

##### Perceived barriers

Perceived barriers to medication adherence also emerged as a decisive factor. Saravanakumar et al. [51], Weng et al. [58], and Zhang et al. [62] found that greater perceived barriers were significantly associated with non-adherence. Similarly, Yang et al. [59] highlighted the role of lower intentions to adhere, consistent with the Theory of Planned Behaviour models.

##### Health literacy

Health literacy, defined as the ability to understand and use health information [66], has been frequently linked to medication non-adherence. Several studies have identified lower health literacy as a significant factor that affects non-adherence [21, 29, 48, 63], whereas others have found no association [27]. These mixed findings likely reflect methodological differences, particularly the use of varied health literacy measurement tools across studies.

##### Acceptance and treatment satisfaction

Zachciał et al. [61] provided additional insight into patients’ beliefs and perspectives by noting the influence of illness acceptance in influencing medication adherence, while several studies [20, 37, 54] found that lower treatment satisfaction with convenience, side effects, and medication information is associated with poorer adherence.

#### Coping strategies

Maladaptive coping strategies, in particular avoidance-oriented coping [49] and palliative coping (religion/fantasy thoughts and emotion-focused coping) [28], were associated with poorer medication adherence. In addition, Liu et al. [40] found that having lower perceived self-efficacy in managing anger-irritation, despondency-distress, and positive affect was also associated with non-adherence.

#### Social and contextual factors

Multiple studies highlight social support as a key factor in promoting adherence to immunosuppressive therapy among kidney transplant recipients. Higher perceived social support has been consistently associated with better adherence, suggesting that emotional and practical assistance helps patients manage long-term medication demands [25]. Higher perceived social support has been consistently associated with better adherence [35], suggesting that emotional and practical assistance helps patients manage long-term medication demands. Supportive relationships can reinforce routine medication-taking behaviours and buffer psychosocial stressors. Evidence also indicates that relationship quality, particularly within couples, plays an important role, with stronger relationships linked to improved adherence [45]. Conversely, low social support has been identified as a significant risk factor for non-adherence [52]. Collectively, these findings emphasise the importance of incorporating psychosocial and relational assessments into post-transplant care to support sustained medication adherence.

#### Qualitative insights

Qualitative studies have provided important insights into the lived experiences of medication adherence among kidney transplant recipients. Low et al. [41] explored Australian recipients’ experiences and found that strict immunosuppressive regimens generated significant psychological stress, driven by fear of missed doses and potential graft failure. Social stigma, limited understanding from others, and disruptions to daily routines further increased the burden, while a strong sense of responsibility for transplant outcomes motivated adherence.

Memory et al. [43] examined post-transplant self-management and identified key facilitators, including internal motivation, strong patient–clinician relationships, and adequate medication knowledge. Barriers included limited health literacy, medication side effects, emotional distress, and difficulties integrating complex regimens into everyday life. Similarly, Muduma et al. [44] found that non-adherence was mainly unintentional, driven by forgetfulness, disruptions to routine, pill burden, and difficulties integrating medication schedules into daily life.

Collectively, these findings highlight how emotional, physical, and practical challenges interact to undermine adherence. They underscore the need for targeted psychoeducation, peer support, and practical tools, such as reminders, to help patients sustain long-term medication adherence.

## Discussion

This systematic review represents the first comprehensive synthesis of psychological factors associated with self-reported medication non-adherence among adult kidney transplant recipients, foregrounding patients’ subjective experiences in line with contemporary patient-reported outcomes research. Across 44 studies, medication non-adherence emerged as a persistent challenge, with prevalence frequently exceeding 50%.

Repeated associations across multiple higher-quality studies were identified between higher psychological distress and poorer adherence, most robustly for depressive symptoms, corroborating earlier reviews [8, 9]. Notably, one study distinguishing intentional from unintentional non-adherence found depressive symptoms to be specifically associated with intentional non-adherence [39], highlighting motivational and cognitive–affective processes rather than forgetfulness alone and underscoring the importance of proactive screening for sub-clinical depression among transplant populations. By contrast, findings related to anxiety were less consistent, likely reflecting heterogeneity in measurement approaches across studies. Anxiety was assessed using instruments with differing conceptual emphases and psychometric properties, potentially obscuring true associations. Evidence from studies using transplant-specific tools, such as the Transplant Effects Questionnaire (TxEQ) [67], suggests that context-sensitive measures may better capture anxiety-related processes relevant to adherence.

Medication beliefs consistently emerged as a key factor affecting adherence, aligning with the Necessity–Concerns Framework (NCF), which posits that adherence is shaped by patients’ perceived medication necessity balanced against concerns about adverse effects [68]. These findings highlight the potential role of motivational interviewing and cognitive behavioural interventions in addressing maladaptive medication beliefs.

Health literacy was also identified as a relevant factor, although interpretation is constrained by measurement variability. Beyond measurement issues, limited health literacy may impair patients’ readiness to engage in complex treatment decisions. Kalantar-Zadeh and Zisman-Ilani [69] argue that shared decision-making frameworks often assume baseline literacy levels that may not exist, particularly among underserved populations, contributing to decision unreadiness and compromised self-management. These findings reinforce the importance of literacy-sensitive, culturally tailored tools that support proactive engagement in self-management and adherence behaviours.

Social support was consistently associated with better adherence, reinforcing findings from prior reviews [8, 9], emphasising its importance as a protective factor. However, most evidence derives from Western contexts, limiting its applicability to non-Western or collectivist settings where sociocultural factors such as filial piety, multigenerational living, family obligations, and beliefs about traditional or complementary medicine may shape adherence differently [70]. These considerations warrant further investigation.

Overall, this review highlights the multifactorial and context-dependent nature of medication non-adherence following kidney transplantation. Psychological distress, medication beliefs, literacy-related factors, and social contexts interact to shape adherence behaviours. Clinically, the findings support routine psychosocial screening using structured tools such as the Stanford Integrated Psychosocial Assessment for Transplant (SIPAT) [71], which systematically evaluates the psychosocial readiness and risk of transplant candidates, may assist clinicians in identifying patients at elevated risk of non-adherence and in tailoring interventions accordingly.

However, effective implementation requires multidisciplinary coordination and sensitivity to resource constraints. Screening should occur at multiple points (pre- and post-transplant) to address evolving psychosocial stressors. Barriers such as limited mental health access and clinic time pressures are especially pronounced in low-resource settings. Applying implementation science frameworks may help adapt interventions to local contexts and support sustainable improvements in post-transplant outcomes. Future studies should integrate such frameworks to enhance real-world impact.

### Limitations

Several limitations warrant consideration. Medication non-adherence was operationalised heterogeneously across studies, with multiple measures differing in conceptual focus, recall periods, and psychometric quality, potentially contributing to inconsistent findings. Although a “less than perfect” adherence definition was applied [3], construct differences remained. Prevalence variability may reflect both true behavioural differences and methodological heterogeneity, and should be interpreted with caution, taking instrument characteristics into account. Reliance on self-reported adherence introduces recall and social desirability biases. Limiting inclusion to English-language and predominantly Western studies may restrict generalisability. While methodological quality was generally acceptable, variability in design and measurement approaches necessitates cautious interpretation of the integrated findings. Furthermore, the predominance of cross-sectional designs precludes determining temporal ordering, and the relationships between adherence and psychological variables may be reciprocal and dynamic rather than unidirectional.

### Future directions

Future research should standardise adherence measures, with clear justification and reporting of the dimensions assessed. Sensitivity or subgroup analyses may address unavoidable heterogeneity. There is a need for future longitudinal and mixed-methods research to better clarify temporal and causal pathways. Beyond identifying correlates of non-adherence, future work should deepen understanding of the reasons underlying non-adherence from patients’ perspectives, including the emotional, cognitive, relational, and contextual processes that shape medication-taking behaviour over time. Such work is essential for informing interventions that not only reduce barriers to adherence but also empower kidney transplant recipients to take an active and confident role in managing their long-term health. Expanding studies to non-Western and underrepresented regions, including Southeast Asia, Africa, and Latin America, is essential to enhance cultural validity, equity, and the relevance of interventions.

## Conclusion

This systematic review synthesises psychological factors linked to self-reported medication non-adherence in adult kidney transplant recipients. Strong evidence supports associations with depressive symptoms and social support, while emerging evidence highlights medication beliefs, health literacy, cognition, and coping. Clinically, the findings underscore the need for integrated psychosocial assessment and targeted, psychologically informed interventions within post-transplant care pathways.

## Supplementary Information

Supplementary Material 1.

Supplementary Material 2.

## Data Availability

The data supporting this systematic review’s findings are extracted from published articles available in public databases. The extracted data and quality assessment scores are available from the corresponding author upon reasonable request.
