# Supplementary material for: Psychological factors associated with medication non-adherence after kidney transplant: a systematic review
Source: BMC Psychol. 2026 Jul 27;14:1524. doi: 10.1186/s40359-026-05233-7 (PMC13689237; doi:10.1186/s40359-026-05233-7)
Supplement: Supplementary file 1 [file 40359_2026_5233_MOESM1_ESM.docx]

**Appendix A: Supplementary File 1**

**Table 1. Keyword Combination and Search Hits**

| **Embase (Elsevier)**  Search hits:  168 | ((('renal transplant*' OR 'kidney transplant*') NEAR/30 (depression OR anxiety OR 'transplant effects' OR regret OR personality OR 'cognitive deficits' OR 'cognitive impairment' OR coping OR 'social support')):ab,ti) AND (adherence:ab,ti OR compliance:ab,ti) |
| --- | --- |
| **Pubmed (NLM)**  Search hits:  213 | (("Kidney Transplantation"[Mesh]) OR (renal transplant*[Title/Abstract] OR kidney transplant*[Title/Abstract])) AND (("Depression"[Mesh] OR "Anxiety"[Mesh] OR "Cognitive Dysfunction"[Mesh] OR "Social Support"[Mesh] OR "Coping Skills"[Mesh]) OR (depression[Title/Abstract] OR anxiety[Title/Abstract] OR transplant effects[Title/Abstract] OR regret[Title/Abstract] OR personality[Title/Abstract] OR cognitive deficits[Title/Abstract] OR cognitive impairment[Title/Abstract] OR coping[Title/Abstract] OR social support[Title/Abstract]))) AND (("Medication Adherence"[Mesh]) OR (adherence[Title/Abstract] OR compliance[Title/Abstract])) |
| **PsycINFO (OvidSP)**  Search hits:  32 | (('renal transplant*' or 'kidney transplant*') and (depression or anxiety or 'transplant effects' or regret or personality or 'cognitive deficits' or 'cognitive impairment' or coping or 'social support')).mp. and (adherence or compliance).m_titl. [mp=title, abstract, heading word, table of contents, key concepts, original title, tests & measures, mesh word] |
| **Scopus (Elsevier)**  Search hits:  123 | TITLE-ABS ( ( "renal transplant*" OR "kidney transplant*" ) W/30 ( depression OR anxiety OR "transplant effects" OR regret OR personality OR "cognitive deficits" OR "cognitive impairment" OR coping OR "social support" ) AND ( adherence OR compliance ) ) |
| **Web of Science (Thomson Reuters)**  Search hits:  113 | TS=(("renal transplant*" OR "kidney transplant*" ) NEAR/30 ( depression OR anxiety OR "transplant effects" OR regret OR personality OR "cognitive deficits" OR "cognitive impairment" OR coping OR "social support") AND ( adherence OR compliance)) |
